# {2-[(*S*)-({2-[(*S*)-1-Benzyl­pyrrolidine-2-carboxamido]phen­yl}(phen­yl)methyl­ene)amino]-4-hydroxy­butanoato-κ^4^ *N*,*N*′,*N*′′,*O*}nickel(II)

**DOI:** 10.1107/S1600536808000949

**Published:** 2008-01-16

**Authors:** Alexander Popkov, Milan Nádvorník, Jozef Kožíšek

**Affiliations:** a Department of Nuclear Medicine and Molecular Imaging, University Medical Center, PO Box 30.001, 9700 RB Groningen, The Netherlands; b Department of General and Inorganic Chemistry, Faculty of Chemical Technology, Pardubice University, 53210 Pardubice, Czech Republic; c Institute of Physical Chemistry and Chemical Physics, Slovak University of Technology, Radlinského 9, SK-812 37 Bratislava, Slovak Republic

## Abstract

The central Ni atom of the title compound, [Ni(C_29_H_29_N_3_O_4_)], is coordinated by three N atoms [Ni—N = 1.955 (2), 1.844 (2) and 1.872 (2) Å] and by one O atom [Ni—O = 1.862 (2) Å] in a pseudo-square-planar geometry. The conformation of the hydroxy­butanoate side chain is controlled by a strong intra­molecular hydrogen bond (H⋯O = 1.84 Å).

## Related literature

For related literature, see: Belokon (1992 ▶); Belokon *et al.* (1988 ▶); Carducci *et al.* (2006 ▶); Chung *et al.* (1993 ▶); Gu *et al.* (2004 ▶); Jirman & Popkov (1995 ▶); Jirman *et al.* (1998 ▶); Kožíšek *et al.* (2004 ▶); Langer *et al.* (2007 ▶); Nádvorník & Popkov (2002 ▶); Popkov *et al.* (2003 ▶, 2005 ▶, and references therein).
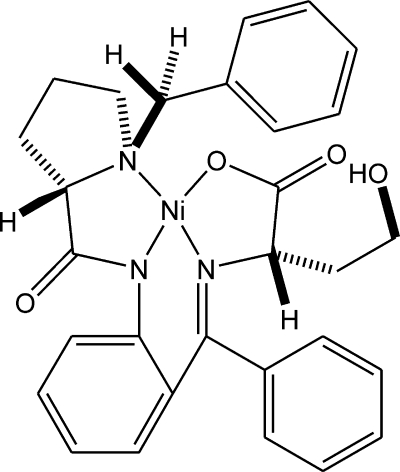


## Experimental

### 

#### Crystal data

[Ni(C_29_H_29_N_3_O_4_)]
*M* *_r_* = 542.26
Orthorhombic, 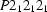

*a* = 9.743 (1) Å
*b* = 10.222 (1) Å
*c* = 26.016 (1) Å
*V* = 2591.0 (4) Å^3^
*Z* = 4
Mo *K*α radiation
μ = 0.79 mm^−1^
*T* = 100 (2) K
0.25 × 0.19 × 0.16 mm

#### Data collection

Oxford Diffraction Gemini R CCD diffractometer
Absorption correction: analytical (Clark & Reid, 1995 ▶) *T*_min_ = 0.840, *T*_max_ = 0.897
62972 measured reflections
5273 independent reflections
4968 reflections with *I* > 2σ(*I*)
*R*_int_ = 0.038

#### Refinement

*R*[*F* ^2^ > 2σ(*F* ^2^)] = 0.029
*wR*(*F* ^2^) = 0.073
*S* = 1.08
5273 reflections
337 parameters
112 restraints
H atoms treated by a mixture of independent and constrained refinement
Δρ_max_ = 0.81 e Å^−3^
Δρ_min_ = −0.29 e Å^−3^
Absolute structure: (Flack, 1983 ▶), 2260 Friedel pairs
Flack parameter: 0.04 (1)

### 

Data collection: *CrysAlis CCD* (Oxford Diffraction, 2006 ▶); cell refinement: *CrysAlis CCD*; data reduction: *CrysAlis RED* (Oxford Diffraction, 2006 ▶); program(s) used to solve structure: *SHELXS97* (Sheldrick, 2008 ▶); program(s) used to refine structure: *SHELXL97* (Sheldrick, 2008 ▶); molecular graphics: *DIAMOND* (Brandenburg, 1998 ▶); software used to prepare material for publication: *enCIFer* (Allen *et al.*, 2004 ▶).

## Supplementary Material

Crystal structure: contains datablocks global, I. DOI: 10.1107/S1600536808000949/sg2215sup1.cif

Structure factors: contains datablocks I. DOI: 10.1107/S1600536808000949/sg2215Isup2.hkl

Additional supplementary materials:  crystallographic information; 3D view; checkCIF report

## Figures and Tables

**Table 1:** Hydrogen-bond geometry (Å, °)

*D*—H⋯*A*	*D*—H	H⋯*A*	*D*⋯*A*	*D*—H⋯*A*
O4—H4*W*⋯O3	0.950 (7)	1.840 (8)	2.726 (3)	154.0 (9)
C7—H7*A*⋯O1	0.95	2.26	2.837 (3)	118
C1—H1*B*⋯O2	0.99	2.31	2.879 (3)	115

**Fig. 1.**
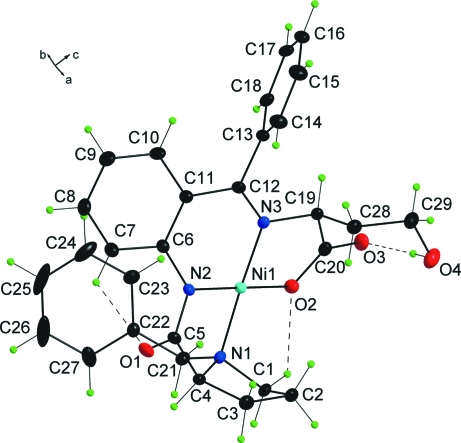
The molecular structure of I, with the numbering scheme of the molecule. Displacement ellipsoids are drawn at the 30% probability level. Hydrogen-bond is indicated by dashed line.

## supplementary crystallographic information

### Comment

Ni^II^ complexes of Schiff bases of *(S)—N*-(2-benzoylphenyl)-1-benzylpyrrolidine-2-carboxamide and α-amino acids are frequently used as chiral α-amino acids synthons in preparative asymmetric syntheses of non-proteinogenic α-amino acids (Belokon *et al.*, 1992; Popkov *et al.*, 2005). One of the most unique applications is syntheses of enantiomerically pure α-imino acids which are of great importance in design of conformationally restricted peptidomimetics (Belokon *et al.*, 1988; Chung *et al.*, 1993). X-ray structures of intermediate complexes bearing a hydroxy group in ω-position of the amino acid fragment side chain have not been published. During course of search for chiral nickel(II) complexes suitable for charge-density studies (Kožíšek *et al.*, 2004), we investigated the first representative of this class, *viz*. the Ni^II^ complex of the Schiff base of (*S*)-*N*-(2-benzoylphenyl)-1-benzylpyrrolidine-2-carboxamide and (*S*)-2-amino-4-hydroxybutanoic acid.

The asymmetric unit of the title compound (Fig. 1) contains one molecule. The Ni atom is pseudo-square-planar coordinated by three N atoms [1.955 (2), 1.844 (2) and 1.872 (2) Å] and by one O atom [1.862 (2) Å].

Crystal structure studied could be compared to those ones which differ by substituents in position C(19). In the case if there are no substituents (Ni^II^ complex of the Schiff base of *(S)—N*-(2-benzoylphenyl)-1-benzylpyrrolidine-2-carboxamide and glycine), the complex has in average 0.022 Å shorter Ni—N and Ni—O distances due to lower steric hindrance [Popkov *et al.*, 2003].

A very similar complex to the studied one, bearing *(S)*-2-aminohept-6-enoic acid residue which does not form the hydrogen bond with O3 as *(S)*-2-amino-4-hydroxybutanoic acid does, also have shorter Ni—N distances (Ni—N1 1.941 Å, Ni—N2 1.845 Å, Ni—N3 1.862 Å, Ni—O4 1.861 Å and Ni—N1 1.955 Å, Ni—N2 1.844 Å, Ni—N3 1.872 Å, Ni—O2 1.862 Å, respectively), but the difference is not statistically significant [Carducci *et al.*, 2006]. The difference can be attributed to not so strong distortion of the amino acid residue ring and distortion of the whole complex due to lack of the intramolecular hydrogen bond.

The most sterically hindered complexes derived from α-quaternary α-amino acids demonstrate similar average Ni—N and Ni—O distances as the studied compound (the Ni^II^ complex of the Schiff base of (*S*)-*N*-(2-benzoylphenyl)-1-benzylpyrrolidine-2-carboxamide and (*S*)-2-amino-2-methylhex-5-enoic acid (Gu *et al.*, 2004) and the Ni^II^ complex of the Schiff base of (*S*)-*N*-(2-benzoylphenyl)-1-benzylpyrrolidine-2-carboxamide and 2-amino-2-methyl-propanoic acid (Langer *et al.*, 2007).

Subsequent addition of the substituents has similar effect to distances of the benzyl groups from the nickel atoms. In the non-substituted complex [Popkov *et al.*, 2003] the distance Ni—C22 is the shortest - 2.928 Å; in the monosubstituted it is half-angstrom longer [Ni—C22 3.431 and 3.467 Å (due to disorder) Carducci, *et al.*, 2006], and in both bis-substituted [Gu *et al.*, 2004 and Langer *et al.*, 2007] the distances are third-angstrom longer (3.268 and 3.337 Å, respectively). The distances of the benzyl groups from the nickel atoms should be similar in deuterochloroform solutions; in NMR spectra of the complexes a number of unique long-range spin-spin interactions and NOE interactions were observed for the Ni^II^ complex of the Schiff base of *(S)—N*-(2-benzoylphenyl)-1-benzylpyrrolidine-2-carboxamide and glycine, but not for the Ni^II^ complex of the Schiff base of *(S)—N*-(2-benzoylphenyl)-1-benzyl-pyrrolidine-2-carboxamide and 2-amino-2-methylpropanoic acid [Jirman & Popkov, 1995, Jirman *et al.*, 1998, Popkov *et al.*, 2003, Langer *et al.*, 2007].

Interesting feature of the crystal structure is a strong intramolecular hydrogen bond O4—H4AW···O3 (153.7 °) (Table 2, Fig.1), which controls the conformation of a hydroxybutanoic acid side-chain.

### Experimental

Ni^II^ complex of the Schiff base of *(S)—N*-(2-benzoylphenyl)-1- benzylpyrrolidine-2-carboxamide and *(S)*-2-amino-4-hydroxybutanoic acid (*L*-homoserine) was prepared using a standard procedure previously described for a similar complex derived from glycine (Nádvorník, Popkov 2002). Single crystals were grown from acetone solution; the compound was fully characterized by ^1^H-NMR, ^13^C-NMR and tandem MS*^n^* techniques.

### Refinement

(type here to add refinement details)

### Crystal data

**Table:**

[Ni(C_29_H_29_N_3_O_4_)]	*F*(000) = 1136
*M**_r_* = 542.26	*D*_x_ = 1.390 Mg m^−^^3^
Orthorhombic, *P*2_1_2_1_2_1_	Mo *K*α radiation, λ = 0.71073 Å
Hall symbol: P 2ac 2ab	Cell parameters from 35886 reflections
*a* = 9.743 (1) Å	θ = 3.2–35.3°
*b* = 10.222 (1) Å	µ = 0.79 mm^−^^1^
*c* = 26.016 (1) Å	*T* = 100 K
*V* = 2591.0 (4) Å^3^	Block, orange
*Z* = 4	0.25 × 0.19 × 0.16 mm

### Data collection

**Table:**

Oxford Diffraction Gemini R CCD diffractometer	5273 independent reflections
Radiation source: fine-focus sealed tube	4968 reflections with *I* > 2σ(*I*)
graphite	*R*_int_ = 0.038
Rotation method data acquisition using ω and φ scans	θ_max_ = 26.4°, θ_min_ = 4.2°
Absorption correction: analytical (Clark & Reid, 1995)	*h* = −12→12
*T*_min_ = 0.840, *T*_max_ = 0.897	*k* = −12→12
62972 measured reflections	*l* = −32→32

### Refinement

**Table:**

Refinement on *F*^2^	Secondary atom site location: difference Fourier map
Least-squares matrix: full	Hydrogen site location: inferred from neighbouring sites
*R*[*F*^2^ > 2σ(*F*^2^)] = 0.029	H atoms treated by a mixture of independent and constrained refinement
*wR*(*F*^2^) = 0.073	*w* = 1/[σ^2^(*F*_o_^2^) + (0.0343*P*)^2^ + 1.651*P*] where *P* = (*F*_o_^2^ + 2*F*_c_^2^)/3
*S* = 1.08	(Δ/σ)_max_ < 0.001
5273 reflections	Δρ_max_ = 0.81 e Å^−^^3^
337 parameters	Δρ_min_ = −0.29 e Å^−^^3^
112 restraints	Absolute structure: (Flack, 1983), 2260 Friedel pairs
Primary atom site location: structure-invariant direct methods	Flack parameter: 0.04 (1)

### Special details

**Table:**

Experimental. face-indexed (*CrysAlis RED*; Oxford Diffraction, 2006)
Geometry. All e.s.d.'s (except the e.s.d. in the dihedral angle between two l.s. planes) are estimated using the full covariance matrix. The cell e.s.d.'s are taken into account individually in the estimation of e.s.d.'s in distances, angles and torsion angles; correlations between e.s.d.'s in cell parameters are only used when they are defined by crystal symmetry. An approximate (isotropic) treatment of cell e.s.d.'s is used for estimating e.s.d.'s involving l.s. planes.
Refinement. Refinement of *F*^2^ against ALL reflections. The weighted *R*-factor *wR* and goodness of fit *S* are based on *F*^2^, conventional *R*-factors *R* are based on *F*, with *F* set to zero for negative *F*^2^. The threshold expression of *F*^2^ > σ(*F*^2^) is used only for calculating *R*-factors(gt) *etc*. and is not relevant to the choice of reflections for refinement. *R*-factors based on *F*^2^ are statistically about twice as large as those based on *F*, and *R*- factors based on ALL data will be even larger.

### Fractional atomic coordinates and isotropic or equivalent isotropic displacement parameters (Å^2^)

**Table:**

	*x*	*y*	*z*	*U*_iso_*/*U*_eq_	
C1	1.1251 (2)	0.7826 (2)	0.09762 (9)	0.0266 (5)	
H1B	1.1797	0.8305	0.1236	0.032*	
H1A	1.1836	0.7650	0.0673	0.032*	
C2	1.0675 (3)	0.6558 (3)	0.11981 (10)	0.0298 (6)	
H2B	1.0323	0.6690	0.1551	0.036*	
H2A	1.1378	0.5859	0.1202	0.036*	
C3	0.9499 (3)	0.6230 (2)	0.08195 (10)	0.0292 (5)	
H3B	0.8700	0.5860	0.1005	0.035*	
H3A	0.9808	0.5593	0.0557	0.035*	
C4	0.9116 (3)	0.7574 (2)	0.05630 (8)	0.0212 (5)	
H4A	0.9335	0.7550	0.0188	0.025*	
C5	0.7633 (2)	0.7917 (2)	0.06404 (8)	0.0207 (5)	
C6	0.6196 (2)	0.9454 (2)	0.11106 (8)	0.0191 (4)	
C7	0.5289 (2)	0.9622 (3)	0.06902 (9)	0.0249 (5)	
H7A	0.5496	0.9228	0.0369	0.030*	
C8	0.4110 (3)	1.0349 (3)	0.07429 (10)	0.0307 (5)	
H8A	0.3517	1.0467	0.0457	0.037*	
C9	0.3781 (3)	1.0920 (3)	0.12203 (10)	0.0358 (6)	
H9A	0.2956	1.1408	0.1254	0.043*	
C10	0.4646 (2)	1.0779 (3)	0.16439 (10)	0.0298 (5)	
H10A	0.4405	1.1165	0.1964	0.036*	
C11	0.5877 (2)	1.0065 (2)	0.15983 (8)	0.0208 (5)	
C12	0.6754 (2)	1.0000 (2)	0.20620 (9)	0.0200 (5)	
C13	0.6158 (3)	1.0400 (2)	0.25796 (8)	0.0220 (5)	
C14	0.5219 (3)	0.9572 (3)	0.28276 (10)	0.0327 (6)	
H14A	0.4997	0.8746	0.2682	0.039*	
C15	0.4606 (3)	0.9970 (3)	0.32928 (11)	0.0388 (7)	
H15A	0.3980	0.9401	0.3461	0.047*	
C16	0.4906 (3)	1.1192 (3)	0.35113 (10)	0.0371 (6)	
H16A	0.4479	1.1456	0.3823	0.045*	
C17	0.5839 (3)	1.2014 (3)	0.32650 (10)	0.0318 (6)	
H17A	0.6053	1.2842	0.3410	0.038*	
C18	0.6471 (2)	1.1622 (3)	0.28005 (10)	0.0267 (5)	
H18A	0.7109	1.2186	0.2636	0.032*	
C19	0.8890 (3)	0.9518 (2)	0.24976 (8)	0.0210 (4)	
H19A	0.8642	1.0219	0.2749	0.025*	
C20	1.0355 (3)	0.9710 (2)	0.23118 (9)	0.0239 (5)	
C21	1.0390 (2)	0.9727 (2)	0.04879 (10)	0.0237 (5)	
H21B	1.0992	1.0318	0.0688	0.028*	
H21A	1.0936	0.9389	0.0196	0.028*	
C22	0.9215 (2)	1.0526 (2)	0.02739 (9)	0.0204 (5)	
C23	0.8585 (3)	1.1494 (2)	0.05785 (10)	0.0280 (5)	
H23A	0.8897	1.1659	0.0918	0.034*	
C24	0.7494 (3)	1.2206 (3)	0.03723 (15)	0.0481 (8)	
H24A	0.7042	1.2847	0.0575	0.058*	
C25	0.7059 (3)	1.1975 (3)	−0.01382 (18)	0.0618 (11)	
H25A	0.6310	1.2457	−0.0275	0.074*	
C26	0.7713 (4)	1.1055 (3)	−0.04396 (15)	0.0617 (11)	
H26A	0.7433	1.0926	−0.0786	0.074*	
C27	0.8778 (4)	1.0321 (3)	−0.02365 (10)	0.0393 (7)	
H27A	0.9217	0.9677	−0.0442	0.047*	
C28	0.8688 (3)	0.8146 (2)	0.27477 (9)	0.0287 (6)	
H28B	0.7705	0.8042	0.2836	0.034*	
H28A	0.8915	0.7470	0.2489	0.034*	
C29	0.9549 (3)	0.7881 (3)	0.32357 (11)	0.0346 (6)	
H29B	0.9454	0.8638	0.3470	0.041*	
H29A	0.9167	0.7106	0.3413	0.041*	
N1	0.99995 (19)	0.8581 (2)	0.08284 (7)	0.0204 (4)	
N2	0.7433 (2)	0.87954 (18)	0.10419 (7)	0.0184 (4)	
N3	0.80154 (19)	0.96293 (18)	0.20289 (7)	0.0181 (4)	
Ni1	0.89673 (3)	0.91512 (3)	0.143270 (10)	0.01780 (8)	
O1	0.67597 (18)	0.73706 (18)	0.03689 (6)	0.0271 (4)	
O2	1.05357 (17)	0.95424 (18)	0.18116 (7)	0.0261 (4)	
O3	1.12652 (18)	0.99616 (19)	0.26255 (7)	0.0324 (4)	
O4	1.0957 (2)	0.7663 (2)	0.31439 (8)	0.0480 (5)	
H4W	1.1334 (14)	0.8443 (19)	0.3004 (11)	0.058*	

### Atomic displacement parameters (Å^2^)

**Table:**

	*U*^11^	*U*^22^	*U*^33^	*U*^12^	*U*^13^	*U*^23^
C1	0.0218 (12)	0.0316 (13)	0.0265 (11)	0.0112 (10)	−0.0007 (9)	0.0017 (10)
C2	0.0376 (15)	0.0261 (13)	0.0258 (12)	0.0120 (11)	0.0002 (10)	0.0024 (10)
C3	0.0357 (13)	0.0230 (13)	0.0290 (12)	0.0036 (10)	0.0032 (10)	0.0033 (10)
C4	0.0257 (12)	0.0194 (11)	0.0186 (10)	0.0041 (10)	0.0031 (9)	0.0000 (8)
C5	0.0244 (11)	0.0196 (11)	0.0180 (10)	−0.0018 (9)	0.0023 (9)	−0.0010 (9)
C6	0.0163 (10)	0.0217 (11)	0.0192 (10)	−0.0045 (9)	0.0020 (8)	0.0020 (8)
C7	0.0169 (11)	0.0349 (13)	0.0229 (11)	−0.0022 (10)	0.0021 (9)	0.0009 (10)
C8	0.0173 (11)	0.0444 (15)	0.0304 (12)	0.0019 (11)	−0.0059 (10)	0.0041 (11)
C9	0.0219 (12)	0.0484 (16)	0.0372 (13)	0.0101 (13)	−0.0022 (10)	−0.0050 (13)
C10	0.0180 (11)	0.0410 (15)	0.0305 (12)	0.0064 (12)	0.0012 (9)	−0.0086 (12)
C11	0.0172 (11)	0.0230 (11)	0.0221 (10)	−0.0020 (9)	0.0004 (9)	−0.0020 (8)
C12	0.0201 (11)	0.0187 (11)	0.0213 (11)	−0.0022 (9)	0.0017 (9)	−0.0030 (9)
C13	0.0205 (11)	0.0260 (11)	0.0194 (10)	0.0033 (10)	−0.0005 (9)	−0.0048 (8)
C14	0.0342 (14)	0.0338 (14)	0.0300 (13)	−0.0007 (11)	0.0077 (11)	−0.0042 (11)
C15	0.0359 (15)	0.0488 (18)	0.0317 (14)	0.0007 (13)	0.0116 (12)	−0.0010 (13)
C16	0.0351 (14)	0.0528 (17)	0.0234 (12)	0.0185 (12)	0.0014 (11)	−0.0074 (12)
C17	0.0257 (13)	0.0383 (14)	0.0313 (13)	0.0151 (11)	−0.0080 (10)	−0.0153 (11)
C18	0.0213 (12)	0.0271 (13)	0.0318 (13)	0.0071 (10)	−0.0047 (10)	−0.0057 (10)
C19	0.0225 (11)	0.0204 (11)	0.0201 (10)	0.0026 (10)	−0.0030 (9)	−0.0032 (8)
C20	0.0238 (12)	0.0206 (12)	0.0272 (12)	0.0015 (10)	−0.0034 (10)	0.0004 (9)
C21	0.0180 (11)	0.0256 (12)	0.0277 (12)	0.0014 (10)	0.0054 (10)	0.0042 (10)
C22	0.0187 (11)	0.0196 (11)	0.0229 (10)	−0.0043 (8)	0.0001 (8)	0.0047 (8)
C23	0.0295 (14)	0.0213 (12)	0.0333 (13)	0.0010 (10)	0.0065 (10)	0.0029 (10)
C24	0.0300 (14)	0.0269 (15)	0.088 (2)	0.0076 (12)	0.0157 (16)	0.0203 (15)
C25	0.0363 (17)	0.0403 (19)	0.109 (3)	−0.0102 (14)	−0.0359 (19)	0.040 (2)
C26	0.085 (3)	0.0305 (18)	0.069 (2)	−0.0197 (17)	−0.054 (2)	0.0208 (15)
C27	0.065 (2)	0.0250 (13)	0.0283 (13)	−0.0059 (14)	−0.0126 (14)	0.0033 (10)
C28	0.0347 (15)	0.0225 (12)	0.0289 (12)	0.0039 (10)	0.0014 (10)	0.0015 (10)
C29	0.0317 (13)	0.0392 (16)	0.0328 (14)	0.0095 (12)	0.0071 (11)	0.0141 (12)
N1	0.0174 (9)	0.0215 (10)	0.0221 (9)	0.0029 (8)	0.0009 (7)	0.0032 (8)
N2	0.0185 (9)	0.0221 (10)	0.0148 (8)	0.0003 (7)	0.0016 (7)	0.0009 (7)
N3	0.0172 (9)	0.0166 (9)	0.0205 (9)	−0.0007 (7)	−0.0017 (7)	−0.0011 (7)
Ni1	0.01522 (12)	0.02042 (13)	0.01776 (12)	0.00077 (11)	0.00044 (11)	−0.00103 (11)
O1	0.0277 (9)	0.0332 (10)	0.0204 (8)	−0.0057 (8)	0.0002 (7)	−0.0052 (7)
O2	0.0178 (8)	0.0332 (10)	0.0272 (8)	0.0001 (7)	−0.0009 (7)	−0.0035 (7)
O3	0.0254 (10)	0.0390 (11)	0.0328 (9)	−0.0015 (8)	−0.0097 (8)	−0.0034 (8)
O4	0.0386 (11)	0.0538 (13)	0.0516 (12)	0.0148 (11)	0.0041 (11)	0.0190 (10)

### Geometric parameters (Å, °)

**Table:**

C1—N1	1.494 (3)	C16—H16A	0.9500
C1—C2	1.526 (4)	C17—C18	1.414 (3)
C1—H1B	0.9900	C17—H17A	0.9500
C1—H1A	0.9900	C18—H18A	0.9500
C2—C3	1.548 (4)	C19—N3	1.492 (3)
C2—H2B	0.9900	C19—C20	1.519 (3)
C2—H2A	0.9900	C19—C28	1.559 (3)
C3—C4	1.573 (3)	C19—H19A	1.0000
C3—H3B	0.9900	C20—O3	1.232 (3)
C3—H3A	0.9900	C20—O2	1.324 (3)
C4—C5	1.501 (3)	C21—C22	1.512 (3)
C4—N1	1.509 (3)	C21—N1	1.517 (3)
C4—H4A	1.0000	C21—H21B	0.9900
C5—O1	1.239 (3)	C21—H21A	0.9900
C5—N2	1.391 (3)	C22—C23	1.408 (3)
C6—N2	1.392 (3)	C22—C27	1.410 (3)
C6—C7	1.417 (3)	C23—C24	1.396 (4)
C6—C11	1.448 (3)	C23—H23A	0.9500
C7—C8	1.374 (4)	C24—C25	1.414 (6)
C7—H7A	0.9500	C24—H24A	0.9500
C8—C9	1.409 (4)	C25—C26	1.380 (6)
C8—H8A	0.9500	C25—H25A	0.9500
C9—C10	1.395 (4)	C26—C27	1.386 (5)
C9—H9A	0.9500	C26—H26A	0.9500
C10—C11	1.409 (3)	C27—H27A	0.9500
C10—H10A	0.9500	C28—C29	1.546 (4)
C11—C12	1.480 (3)	C28—H28B	0.9900
C12—N3	1.289 (3)	C28—H28A	0.9900
C12—C13	1.522 (3)	C29—O4	1.409 (4)
C13—C14	1.403 (4)	C29—H29B	0.9900
C13—C18	1.408 (3)	C29—H29A	0.9900
C14—C15	1.409 (4)	N1—Ni1	1.9552 (19)
C14—H14A	0.9500	N2—Ni1	1.8439 (19)
C15—C16	1.404 (4)	N3—Ni1	1.8721 (19)
C15—H15A	0.9500	Ni1—O2	1.8619 (17)
C16—C17	1.393 (4)	O4—H4W	0.950 (7)
			
N1—C1—C2	103.65 (19)	N3—C19—C20	105.45 (18)
N1—C1—H1B	111.0	N3—C19—C28	109.73 (19)
C2—C1—H1B	111.0	C20—C19—C28	111.6 (2)
N1—C1—H1A	111.0	N3—C19—H19A	110.0
C2—C1—H1A	111.0	C20—C19—H19A	110.0
H1B—C1—H1A	109.0	C28—C19—H19A	110.0
C1—C2—C3	102.46 (19)	O3—C20—O2	125.6 (2)
C1—C2—H2B	111.3	O3—C20—C19	119.5 (2)
C3—C2—H2B	111.3	O2—C20—C19	114.9 (2)
C1—C2—H2A	111.3	C22—C21—N1	116.21 (19)
C3—C2—H2A	111.3	C22—C21—H21B	108.2
H2B—C2—H2A	109.2	N1—C21—H21B	108.2
C2—C3—C4	104.8 (2)	C22—C21—H21A	108.2
C2—C3—H3B	110.8	N1—C21—H21A	108.2
C4—C3—H3B	110.8	H21B—C21—H21A	107.4
C2—C3—H3A	110.8	C23—C22—C27	120.2 (2)
C4—C3—H3A	110.8	C23—C22—C21	120.1 (2)
H3B—C3—H3A	108.9	C27—C22—C21	119.7 (2)
C5—C4—N1	109.19 (18)	C24—C23—C22	118.8 (3)
C5—C4—C3	112.0 (2)	C24—C23—H23A	120.6
N1—C4—C3	105.49 (18)	C22—C23—H23A	120.6
C5—C4—H4A	110.0	C23—C24—C25	120.1 (3)
N1—C4—H4A	110.0	C23—C24—H24A	119.9
C3—C4—H4A	110.0	C25—C24—H24A	119.9
O1—C5—N2	128.5 (2)	C26—C25—C24	120.6 (3)
O1—C5—C4	118.7 (2)	C26—C25—H25A	119.7
N2—C5—C4	112.71 (19)	C24—C25—H25A	119.7
N2—C6—C7	119.99 (19)	C25—C26—C27	119.9 (3)
N2—C6—C11	120.46 (19)	C25—C26—H26A	120.1
C7—C6—C11	119.4 (2)	C27—C26—H26A	120.1
C8—C7—C6	120.6 (2)	C26—C27—C22	120.3 (3)
C8—C7—H7A	119.7	C26—C27—H27A	119.8
C6—C7—H7A	119.7	C22—C27—H27A	119.8
C7—C8—C9	120.2 (2)	C29—C28—C19	115.6 (2)
C7—C8—H8A	119.9	C29—C28—H28B	108.4
C9—C8—H8A	119.9	C19—C28—H28B	108.4
C10—C9—C8	121.0 (2)	C29—C28—H28A	108.4
C10—C9—H9A	119.5	C19—C28—H28A	108.4
C8—C9—H9A	119.5	H28B—C28—H28A	107.5
C9—C10—C11	120.1 (2)	O4—C29—C28	114.6 (2)
C9—C10—H10A	120.0	O4—C29—H29B	108.6
C11—C10—H10A	120.0	C28—C29—H29B	108.6
C10—C11—C6	118.7 (2)	O4—C29—H29A	108.6
C10—C11—C12	116.5 (2)	C28—C29—H29A	108.6
C6—C11—C12	124.8 (2)	H29B—C29—H29A	107.6
N3—C12—C11	120.6 (2)	C1—N1—C4	103.35 (18)
N3—C12—C13	120.1 (2)	C1—N1—C21	110.15 (18)
C11—C12—C13	119.3 (2)	C4—N1—C21	113.74 (18)
C14—C13—C18	119.2 (2)	C1—N1—Ni1	111.52 (14)
C14—C13—C12	119.5 (2)	C4—N1—Ni1	106.14 (13)
C18—C13—C12	121.1 (2)	C21—N1—Ni1	111.62 (15)
C13—C14—C15	119.8 (3)	C5—N2—C6	121.98 (19)
C13—C14—H14A	120.1	C5—N2—Ni1	115.33 (15)
C15—C14—H14A	120.1	C6—N2—Ni1	122.40 (15)
C16—C15—C14	121.1 (3)	C12—N3—C19	120.82 (19)
C16—C15—H15A	119.5	C12—N3—Ni1	127.19 (16)
C14—C15—H15A	119.5	C19—N3—Ni1	111.98 (14)
C17—C16—C15	119.1 (2)	N2—Ni1—O2	178.29 (8)
C17—C16—H16A	120.5	N2—Ni1—N3	96.12 (8)
C15—C16—H16A	120.5	O2—Ni1—N3	84.94 (8)
C16—C17—C18	120.4 (2)	N2—Ni1—N1	85.11 (8)
C16—C17—H17A	119.8	O2—Ni1—N1	93.88 (8)
C18—C17—H17A	119.8	N3—Ni1—N1	177.24 (9)
C13—C18—C17	120.4 (2)	C20—O2—Ni1	116.03 (16)
C13—C18—H18A	119.8	C29—O4—H4W	108.0 (13)
C17—C18—H18A	119.8		
			
N1—C1—C2—C3	41.8 (2)	C2—C1—N1—C21	−166.68 (19)
C1—C2—C3—C4	−22.6 (2)	C2—C1—N1—Ni1	68.8 (2)
C2—C3—C4—C5	−122.5 (2)	C5—C4—N1—C1	150.08 (18)
C2—C3—C4—N1	−3.8 (2)	C3—C4—N1—C1	29.5 (2)
N1—C4—C5—O1	165.5 (2)	C5—C4—N1—C21	−90.5 (2)
C3—C4—C5—O1	−78.0 (3)	C3—C4—N1—C21	148.95 (19)
N1—C4—C5—N2	−17.5 (2)	C5—C4—N1—Ni1	32.61 (19)
C3—C4—C5—N2	99.0 (2)	C3—C4—N1—Ni1	−87.93 (17)
N2—C6—C7—C8	−175.3 (2)	C22—C21—N1—C1	175.5 (2)
C11—C6—C7—C8	−0.5 (4)	C22—C21—N1—C4	60.1 (3)
C6—C7—C8—C9	−1.0 (4)	C22—C21—N1—Ni1	−60.0 (2)
C7—C8—C9—C10	1.0 (4)	O1—C5—N2—C6	−17.3 (4)
C8—C9—C10—C11	0.5 (5)	C4—C5—N2—C6	166.07 (19)
C9—C10—C11—C6	−1.9 (4)	O1—C5—N2—Ni1	168.8 (2)
C9—C10—C11—C12	178.2 (3)	C4—C5—N2—Ni1	−7.9 (2)
N2—C6—C11—C10	176.8 (2)	C7—C6—N2—C5	−22.5 (3)
C7—C6—C11—C10	1.9 (3)	C11—C6—N2—C5	162.7 (2)
N2—C6—C11—C12	−3.3 (3)	C7—C6—N2—Ni1	151.03 (18)
C7—C6—C11—C12	−178.2 (2)	C11—C6—N2—Ni1	−23.8 (3)
C10—C11—C12—N3	−164.5 (2)	C11—C12—N3—C19	−177.90 (19)
C6—C11—C12—N3	15.6 (3)	C13—C12—N3—C19	3.3 (3)
C10—C11—C12—C13	14.3 (3)	C11—C12—N3—Ni1	1.0 (3)
C6—C11—C12—C13	−165.6 (2)	C13—C12—N3—Ni1	−177.85 (16)
N3—C12—C13—C14	−107.6 (3)	C20—C19—N3—C12	−153.7 (2)
C11—C12—C13—C14	73.5 (3)	C28—C19—N3—C12	85.9 (3)
N3—C12—C13—C18	76.1 (3)	C20—C19—N3—Ni1	27.2 (2)
C11—C12—C13—C18	−102.7 (3)	C28—C19—N3—Ni1	−93.10 (19)
C18—C13—C14—C15	−0.2 (4)	C5—N2—Ni1—O2	76 (3)
C12—C13—C14—C15	−176.5 (2)	C6—N2—Ni1—O2	−98 (3)
C13—C14—C15—C16	0.8 (4)	C5—N2—Ni1—N3	−154.96 (16)
C14—C15—C16—C17	−0.8 (4)	C6—N2—Ni1—N3	31.13 (17)
C15—C16—C17—C18	0.2 (4)	C5—N2—Ni1—N1	22.57 (16)
C14—C13—C18—C17	−0.4 (4)	C6—N2—Ni1—N1	−151.35 (17)
C12—C13—C18—C17	175.9 (2)	C12—N3—Ni1—N2	−20.3 (2)
C16—C17—C18—C13	0.3 (4)	C19—N3—Ni1—N2	158.62 (15)
N3—C19—C20—O3	163.7 (2)	C12—N3—Ni1—O2	158.3 (2)
C28—C19—C20—O3	−77.2 (3)	C19—N3—Ni1—O2	−22.72 (15)
N3—C19—C20—O2	−18.5 (3)	C12—N3—Ni1—N1	−136.9 (18)
C28—C19—C20—O2	100.6 (2)	C19—N3—Ni1—N1	42.1 (19)
N1—C21—C22—C23	81.3 (3)	C1—N1—Ni1—N2	−142.28 (16)
N1—C21—C22—C27	−100.4 (3)	C4—N1—Ni1—N2	−30.41 (14)
C27—C22—C23—C24	2.4 (4)	C21—N1—Ni1—N2	94.03 (16)
C21—C22—C23—C24	−179.4 (2)	C1—N1—Ni1—O2	39.10 (16)
C22—C23—C24—C25	−1.6 (4)	C4—N1—Ni1—O2	150.97 (14)
C23—C24—C25—C26	−0.6 (5)	C21—N1—Ni1—O2	−84.59 (16)
C24—C25—C26—C27	2.0 (5)	C1—N1—Ni1—N3	−25.5 (19)
C25—C26—C27—C22	−1.2 (5)	C4—N1—Ni1—N3	86.4 (18)
C23—C22—C27—C26	−1.0 (4)	C21—N1—Ni1—N3	−149.2 (18)
C21—C22—C27—C26	−179.3 (3)	O3—C20—O2—Ni1	179.3 (2)
N3—C19—C28—C29	179.2 (2)	C19—C20—O2—Ni1	1.6 (3)
C20—C19—C28—C29	62.7 (3)	N2—Ni1—O2—C20	141 (3)
C19—C28—C29—O4	−73.6 (3)	N3—Ni1—O2—C20	12.10 (17)
C2—C1—N1—C4	−44.8 (2)	N1—Ni1—O2—C20	−165.41 (17)

### Hydrogen-bond geometry (Å, °)

**Table:**

*D*—H···*A*	*D*—H	H···*A*	*D*···*A*	*D*—H···*A*
O4—H4W···O3	0.95 (1)	1.84 (1)	2.726 (3)	154.(1)
C7—H7A···O1	0.95	2.26	2.837 (3)	118.
C1—H1B···O2	0.99	2.31	2.879 (3)	115.
